# The Association of Context with Reported Self-Efficacy for Cancer-Preventive Behaviors and Perceived Cancer Risk in U.S. Adults from the Midlife in the United States (MIDUS) Study

**DOI:** 10.3390/ijerph21010062

**Published:** 2024-01-03

**Authors:** Catherine M. Pichardo, Laura A. Dwyer, Rebecca A. Ferrer, April Y. Oh

**Affiliations:** 1 Behavioral Research Program, Division of Cancer Control and Population Sciences, National Cancer Institute, National Institutes of Health, Rockville, MD 20892, USA; rebecca.ferrer@nih.gov; 2 Cape Fox Facilities Services, Manassas, VA 20109, USA; laura.dwyer@nih.gov; 3 Division of Cancer Control and Population Sciences, National Cancer Institute, National Institutes of Health, Rockville, MD 20892, USA; april.oh@nih.gov

**Keywords:** cancer prevention self-efficacy, perceived cancer risk, cancer prevention, MIDUS, neighborhood, social integration, neighborhood trust and safety

## Abstract

**Background:** Cancer is one of the leading causes of death in the United States. It is critical to understand the associations among multilevel determinants of cancer prevention and control behaviors. This study examined associations of neighborhood factors with perceived risk of cancer and self-efficacy for reducing cancer risk. **Methods:** Cross-sectional analyses included 2324 U.S. adults from the Midlife in the U.S. Wave 3. Participants completed surveys of neighborhood environment (perceived neighborhood trust and safety, built environment conditions, social integration), perceived cancer risk and cancer prevention efficacy. Multivariate linear regressions examined associations of neighborhood context with risk perceptions and self-efficacy. **Results:** In the model that adjusted for sociodemographic characteristics, better perceived neighborhood trust and safety were associated with lower perceived cancer risk. In fully adjusted models for sociodemographic characteristics and contextual factors, higher perceptions of neighborhood trust and safety were associated with higher cancer prevention self-efficacy. Perceptions of better built neighborhood conditions and higher social integration were significantly associated with lower perceived cancer risk and higher perceived cancer prevention efficacy. **Conclusions:** Perceptions of neighborhood context may play a role in shaping psychosocial factors such as perceived cancer risk and self-efficacy, even after controlling for robust predictors of these perceptions.

## 1. Introduction

Cancer is one of the leading causes of death in the United States [1]. Ecological views suggest that multilevel determinants of health (i.e., social, genetic, environmental and behavioral processes) influence each other and operate together to contribute to cancer outcomes across the cancer control continuum [2,3,4]. While emerging work has examined neighborhood-level effects on cancer risk and outcomes [5], our understanding of how structural and social determinants impact beliefs about cancer prevention and control behaviors remains poor. This study aimed to address research gaps by examining the associations of individual-level beliefs (cancer-related risk perception and cancer prevention self-efficacy) and perceived neighborhood-level (neighborhood disadvantage) factors known to be impactful for cancer prevention and control behavior and outcomes.

The risk for many types of cancer can be mitigated through behavioral and lifestyle changes [6], and survival can be enhanced through early detection via cancer screenings [7,8]. Two constructs included in health behavior theories that are hypothesized to be important for facilitating behavior change towards cancer prevention are (1) perceptions of cancer risk [9] and (2) self-efficacy [10]. One health behavior theory that prominently features these constructs is the Health Belief Model. In this model, behaviors are hypothesized to be elicited, in part, by several individual-level beliefs, including risk perceptions for experiencing a health outcome and self-efficacy for engaging in a health-related action [11]. In the context of cancer, risk perceptions are beliefs about one’s susceptibility to cancer. Studies have linked perceived cancer risk with intentions to engage in cancer-preventive behaviors and actual enactment of such behaviors (i.e., intentions and behavior change to reduce alcohol consumption) [12]. Perceptions of cancer risk have also been associated with cancer screening [12,13] and HPV vaccination [14]. A recent meta-analysis demonstrated that increasing risk perceptions (including cancer risk perceptions) through interventions has significant effects on improving intentions and preventive behaviors [15]. In the context of cancer, self-efficacy can be defined as the perception that one can take actions to mitigate cancer risk. Studies have found self-efficacy to be related to various cancer-preventive behaviors, including smoking cessation [16], cancer-related information seeking [17], breast self-exams [18], and exercise and diet [19,20]. Although research has examined social cognitive predictors of risk perception [13,21,22,23,24] and self-efficacy, it is important to examine neighborhood variables and other contextual predictors of cancer risk and prevention self-efficacy to promote healthy behaviors. This is also consistent with the Health Belief Model, which posits that there are many factors, including social, contextual, and demographic variables, that influence health beliefs, which in turn impact engagement in and maintenance of health behaviors [11].

### The Environment and Health Beliefs

Individuals may form cancer risk perceptions and self-efficacy beliefs as a response to their physical and sociocultural contexts [25,26]. Individual factors (e.g., psychosocial variables, including self-efficacy and risk perception) and contextual factors (e.g., socioeconomic and interpersonal aspects of one’s neighborhood) are reciprocally related to each other [27], and are both salient for health-related behaviors. Research has reported modest correlations between objective and subjective indicators of neighborhood conditions, suggesting individual experiences with neighborhood factors are diverse, and may vary across exposure to objective neighborhood features, dispositional and cognitive factors, personal experiences and social comparison [28]. Overall, unique subjective experiences with one’s neighborhood socio-cultural and built conditions may influence self-efficacy and risk perception in diverse ways, and these influences may vary by other characteristics, such as race and ethnicity and neighborhood tenure.

Subjective experiences with one’s neighborhood can include perceived inequalities, or the extent to which individuals perceive an unequal distribution of life resources. Perceived inequalities are predicted by neighborhood socioeconomic status and race and ethnicity, and are predictive of well-being, stress and behavior, regardless of objective neighborhood conditions [29,30,31,32]. These associations show the importance of assessing individual neighborhood perceptions, including in relation to individual-level health outcomes. For example, recent evidence of relationships between perceived neighborhood context and intrapersonal processes suggests that chronic stress resulting from exposure to neighborhood disadvantage results in lower levels of internal health locus of control and perceived control over one’s health [33]. In addition, residing in neighborhoods with adverse conditions and scarce resources may limit health-related efficacy beliefs [34,35,36]. Overall, individual neighborhood perceptions may be strong determinants of general health due to the social cognitive pathways by which they operate [30] to influence healthful behaviors.

The purpose of this study is to extend the literature on cancer risk perception, self-efficacy and neighborhood context by exploring the association between perceived neighborhood context and individuals’ perceived cancer risk and self-efficacy for cancer prevention in a national sample of adults. We also examine residential tenure (how long individuals have resided in the same neighborhood) as a potential moderator of exposure to neighborhood contextual factors [37]. We also examined if there were racial and ethnic differences, as the literature has suggested disparities in cancer-related outcomes and in neighborhood health-promoting resources [38].

## 2. Methods

**Participants.** The survey of Midlife in the U.S. (MIDUS), a multi-stage probability sample, provided data for this study [39]. Eligible participants in the MIDUS core national sample were non-institutionalized English-speaking adults aged 25 to 74, residing within the coterminous U.S. and from working telephone banks. Specifically, the national sample comprised participants across four subsamples: (1) a national RDD (random digit dialing/selection) sample (*n* = 3487); (2) oversamples from five metropolitan areas in the U.S. (*n* = 757); (3) siblings of participants from the RDD sample (*n* = 950); and (4) a national RDD sample of twin pairs (*n* = 1914) (see *Description of MIDUS Sample Handbook*, https://www.midus.wisc.edu/ (accessed on 1 August 2022)). Probability oversampling of older adults and men was conducted to meet the research agenda of the MIDUS study. The MIDUS sample closely represented the U.S. population. However, it is important to highlight that participants from the Midwest region, whites and participants with more than a high-school degree were slightly over-represented in the sample [40,41].

Secondary data analysis was conducted using data from the MIDUS III Project 1 (*N* = 3294) and the Milwaukee African American Sample (*N* = 389) surveys conducted in 2013 and 2016, respectively. Analyses were cross-sectional, as due to the structure of the data (assessments ten years apart without information about neighborhood beliefs for any residences in the interim) it is not possible to predict how neighborhood perceptions over the past ten years were associated with current risk perception and self-efficacy. The Milwaukee African American Sample was added to the MIDUS cohort in 2005 (Wave 2) to replenish the MIDUS II and increase the number of racial minorities included in the broader MIDUS study. By Wave 3, participants were aged 39–93 years for the MIDUS III Project 1 and 44–94 years in the Milwaukee African American sample [42]. Survey items in the current study were administered in both the MIDUS III Project 1 and the Milwaukee African American Sample. MIDUS data collection was reviewed and approved by the Education and Social/Behavioral Sciences and the Health Sciences IRBs at the University of Wisconsin–Madison. The study was conducted in accordance with the Declaration of Helsinki and free and informed consent was obtained from all subjects involved in the study.

Only individuals who completed the self-administered questionnaires were asked about their cancer prevention efficacy; individuals who completed the phone interview only or partially completed the questionnaires were excluded from analysis (Project 1 *n* = 381; Milwaukee *n =* 62). Of participants who completed the questionnaires, individuals with a personal history of cancer were not asked about their perceived cancer risk and were excluded from this analysis. Those with a personal history of cancer or with missing data on the cancer history question were also excluded from this analysis (Project 1 *n* = 603; Milwaukee = 45), resulting in a sample of 2592.

### Measures

***Perceived cancer risk (PCR).*** Perceived cancer risk was assessed with two items: “Do you think your risk of getting cancer is higher, lower, or about the same as other men/women your age?” Individuals who reported that their risk was either higher or lower were then asked, “Would you say a lot higher [lower], somewhat higher [lower], or only a little higher [lower]?” The scale combined these two items and ranged from 0 (lowest perceived risk) to 6 (highest perceived risk).

***Self-efficacy for cancer prevention***. Efficacy was assessed by asking participants to indicate their agreement with the following statement: “There are certain things I can do for myself to reduce the risk of cancer” (1 (*Strongly Disagree*) to 7 (*Strongly Agree*)).

***Perceived neighborhood trust and safety.*** Perceived neighborhood trust and safety was assessed using a 4-item subscale developed by Keyes, 1988 [43,44,45]. This scale has been previously utilized with the MIDUS cohort [43,46]. Respondents indicated whether the following statements described their situation a lot (1), some (2), a little (3), or not at all (4): “I feel safe being out alone in my neighborhood at night,” “I feel safe being out alone in my neighborhood during the daytime,” “I could call on a neighbor for help if I needed it” and “People in my neighborhood trust each other.” Items were reverse coded and the mean of the four items was calculated (possible range from 1 to 4), with higher scores indicating greater perceived neighborhood trust and safety. Cronbach’s alpha revealed adequate reliability (alpha = 0.65) in the MIDUS sample. Perceived neighborhood trust and safety were categorized into tertiles based on the sample distribution (i.e., low, moderate, high).

***Social integration.*** Social integration was measured by assessing the extent of agreement (1 (*Agree Strongly*) to 7 (*Disagree Strongly*)) on three items: “I don’t feel I belong to anything I’d call a community,” “I feel close to other people in my community [reverse coded],” and “My community is a source of comfort [reverse coded]” [44]. This scale has been previously used in the MIDUS cohort to examine mental health [47]. For an item with a missing value, the mean value of completed items was imputed. Items were summed into a total score (with a possible range from 3 to 21), with higher scores indicating higher social integration. Cronbach’s alpha revealed adequate reliability (alpha = 0.79) in the MIDUS sample. Scores were not calculated for cases with missing values on all items. Similar to other neighborhood variables, scores were categorized into tertiles based on sample distribution, with higher tertiles reflecting higher social integration.

***Perceived neighborhood built conditions.*** We defined neighborhood-built conditions with a 2-item index that captured the conditions of the built environment. “Buildings and streets in my neighborhood are kept in very good repair” and “My neighborhood is kept clean.” Responses ranged from 1 “A lot” to 4 “Not at all”. These two questions are part of the neighborhood conditions domain identified with confirmatory factor analysis in prior studies [43,48], which additionally included a “feel good about home/neighborhood” item. Given that we were particularly interested in examining perceptions of the structural physical conditions of the neighborhood as a separate construct, we excluded the “feel good about home/neighborhood” item. Additionally, this item conflates perceptions of the home and the neighborhood environment, which may differ. These two items were moderately correlated (Spearmen rho = 0.66, *p* < 0.001) and were reverse coded and summed (possible range from 2 to 8), with high scores indicating better built neighborhood conditions. Scores were categorized into tertiles based on sample distribution.

***Covariates*.** Sociodemographic covariates included age (continuous), sex, education (high school degree or less, some college, college degree, graduate degree), income (<$60,000; $60,000 to $99,999; 100,000+), race and ethnicity (non-Hispanic White, non-Hispanic Black, other (Native American or Alaska Native Aleutian Islander/Eskimo; Asian; Native Hawaiian or Pacific Islander; Hispanic/Latino; other)), history of cancer in the respondent’s immediate family (biological parents, brothers, sisters, or children), marital status (married, other (separated, divorced, widowed, never married)) and residential tenure (<6 years, 6 to 14 years, ≥15 years).

The establishment of relations to others, a dimension of psychological well-being [49], was included as a confounder since previous work has linked neighborhood quality and perceived cancer risk to subjective well-being [24,46]. The establishment of positive relations to others is closely linked to neighborhood-level social participation and integration, which influences one’s perceptions of neighborhood-level social cohesion and attachment [50]. These perceptions are closely tied to neighborhoods’ trust and safety, neighborhood factors examined in this study. Establishment of quality ties to others was assessed using the Positive Relations with others sub-scale from Ryff’s (1989) Scales of Psychological Well-Being [51]. Items included: “Maintaining close relationships has been difficult and frustrating for me,” “People would describe me as a giving person, willing to share my time with others [reverse coded],” and “I have not experienced many warm and trusting relationships with others” (1 (*Agree Strongly*) to 7 (*Disagree Strongly*)). Items were summed (possible range from 3 to 21), with higher scores reflecting higher reported positive relations. Cronbach’s alpha revealed adequate reliability (alpha = 0.62) in the MIDUS sample.

**Statistical analysis.** Multivariate linear regression was used to examine the associations of neighborhood context with individuals’ perceived cancer risk and self-efficacy for cancer prevention. Context was defined and measured by perceived neighborhood trust and safety, perceived built neighborhood conditions and social integration. We examined separate models predicting each of the two outcomes (perceived cancer risk and self-efficacy). The first model examined effects of sociodemographic characteristics (family history of cancer, sex, education level, age, race and ethnicity, positive relations). The second model added neighborhood contextual variables and residential tenure. This approach allowed us to examine the unique explanatory variance of contextual variables, while adjusting for the variance explained by sociodemographic variables. Second, sensitivity analyses were conducted to examine unique effects of each neighborhood contextual factor (i.e., perceived neighborhood trust and safety, perceived built neighborhood conditions and social integration in separate models) predicting perceived cancer risk and self-efficacy separately, adjusted for covariates. Effects modification by residential tenure (<6 years, 6 to 14 years, ≥15 years) and race and ethnicity (non-Hispanic White, non-Hispanic Black, other) was also tested in separate models for each contextual variable, adjusted for sociodemographic characteristics.

Rates of missing data ranged from 0.08% to 1.93% for the following variables: perceived neighborhood trust and safety, perceived built neighborhood conditions, social integration education, reported positive relations, family history of cancer, residential tenure, marital status, perceived cancer risk and prevention efficacy. Despite evidence for differential missingness (Little’s MCAR test *p* < 0.000), a less than 5% level of missingness among study variables should minimally impact results [52], and therefore participants with complete data on these individual-level variables were used for the main analysis (*n* = 2450). A missing indicator was given to income (6.25% missing), race (5.56% missing) and ethnicity to account for missingness.

All analyses were conducted in Stata 17.0 and statistical significance was defined as *p* < 0.05. The statistical significance of interaction terms was accepted at the Bonferroni-adjusted *p* < 0.05.

## 3. Results

Participant characteristics are reported in Table 1. The sample was primarily non-Hispanic White (non-Hispanic White 82.01%, non-Hispanic Black 13.38%; Other 4.6%), and approximately half of participants were female (*n* = 1321). Participants reported a mean age of 62.09 years (SD = 10.74), and 30% had a high school or lower level of education. The mean score for perceived cancer prevention efficacy was 5.71 (SD = 1.33) and 2.87 (SD = 1.32) for perceived cancer risk. Bivariate associations showed that age, sex, education and reported positive relations are associated with perceived cancer risk and prevention efficacy. Family history of cancer and residential tenure are only associated with perceived cancer risk (see Supplementary Table S1). 

### Hierarchical Multiple Regression

Results indicate that 10.4% and 3.8% of the variance in perceived cancer risk and perceived cancer prevention efficacy, respectively, was explained by all predictor variables in the full model. The first model (i.e., demographic variables) explained 9.7% of the variance in perceived cancer risk (*R*^2^ = 0.097, *F* (14, 2435) = 19.75, *p* < 0.001) and 2.0% in perceived cancer prevention efficacy (*R*^2^ = 0.020, *F* (14, 2435) = 4.59, *p* < 0.001). Model 2 added perceived built neighborhood conditions, social integration and residential tenure, and explained 0.7% (Δ*R*^2^ = 0.007, *F* (22, 2427) = 13.88, *p* < 0.001) and 1.8% (Δ*R*^2^ = 0.018, *F* (22, 2427) = 5.36, *p* < 0.001) of the variance in cancer risk and cancer prevention efficacy, respectively.

**Main Effect of Perceived Neighborhood Trust and Safety.** In the model that did not adjust for other contextual factors (i.e., perceived built neighborhood conditions, social integration), higher perceived neighborhood trust and safety were associated with lower perceived cancer risk (middle tertile *B* = −0.12; 95% CI = −0.24, −0.001; highest tertile *B* = −0.17, 95% CI: −0.31, −0.03, compared to the lowest tertile, Supplementary Table S2). This association was not seen in the fully adjusted multiple regression model that included all contextual factors and socio-demographic characteristics, Table 2. In contrast, higher perceptions of neighborhood trust and safety were significantly associated with higher cancer prevention efficacy in the fully adjusted model (highest tertile *B* = 0.22, 95% CI = 0.06, 0.38 compared to the lowest tertile, Table 3), and similar results were found in the models that did not adjust for perceptions of the built environment conditions and social integration (Supplementary Table S2).

**Main Effect of Perceived Built Neighborhood Conditions.** Greater perceived built neighborhood conditions were associated with lower perceived cancer risk (highest tertile *B* = −0.16, 95% CI: −0.28, −0.03) compared to the lowest tertile, Table 2) and higher cancer prevention efficacy (highest tertile *B* = 0.14, 95% CI: 0.01, 0.27, compared to the lowest tertile, Table 3) in fully adjusted models. Similar results were found in the models that did not adjust for perceptions of neighborhood trust and safety and social integration.

**Main Effect of Social Integration.** High social integration was associated with lower perceived cancer risk (highest tertile *B* = −0.16, 95% CI: −0.30, −0.02, compared to the lowest tertile, Table 2) and higher perceived cancer prevention efficacy (middle tertile *B =* 0.20, 95% CI: 0.07, 0.32; highest tertile *B* = 0.28, 95% CI =0.14, 0.43, compared to the lowest tertile, Table 3). Similar results were found in the models that did not adjust for perceived neighborhood trust and safety and perceived built neighborhood conditions.

**Interactions with Residential Tenure and Race and Ethnicity.** Residential tenure and race and ethnicity did not moderate the associations between contextual factors with either perceived cancer risk or perceived cancer prevention efficacy (*p* > 0.05).

## 4. Discussion

Guided by socio-ecologic models of health, this study examined associations among multilevel determinants of health behaviors (i.e., individual and environmental factors) in a nationally representative sample. We found that perceptions of a better neighborhood context (neighborhood trust and safety, built neighborhood conditions and social integration) were associated with lower perceived cancer risk and higher cancer prevention self-efficacy. This study did not find that residential tenure or race and ethnicity moderated the associations between perceived neighborhood characteristics and social integration with either perceived cancer risk or self-efficacy. Results suggest that perceptions of neighborhood context may play a role in shaping psychosocial factors such as perceived cancer risk and self-efficacy, even after controlling for robust predictors of these perceptions (such as sociodemographic variables).

Prior studies have demonstrated the role of the neighborhood context, including access and availability of resources, and neighborhood socioeconomic status, in influencing cancer-preventive behaviors and cancer risk and outcomes across the cancer continuum [5,53,54]. Studies have also found that neighborhood perceptions, separate from objective measures of neighborhood characteristics, influence health behaviors and self-rated health [28]. These data are cross-sectional, and we could not control for biases related to neighborhood selection. For example, it is possible individuals self-select into certain types of neighborhoods based on their individual characteristics [5], and would therefore move between similar neighborhoods.

The results of the present study suggest that individuals residing in neighborhoods they perceive to be of higher trust and safety may report lower perceived cancer risk and higher self-efficacy to mitigate cancer risk. Prior literature suggests that environments characterized by problems of quality, safety or disorder can contribute to feelings of powerlessness [55] and decreases in perceived cancer prevention resources [56]. Perceived environmental characteristics might also influence individuals’ perceptions of sources of cancer risk and their beliefs about the efficacy of health behaviors in the context of these environmental risks [55]. The results of this study suggest that the effects of perceived neighborhood environments on cancer-related beliefs are a potentially rich area for further research and intervention.

Little research has been published to date concerning the relationship between inequality (real or perceived) and self-efficacy. One study showed that high proportions of neighborhood unemployment and public assistance were associated with low levels of self-efficacy above and beyond individual-level SES [36]. It has been hypothesized that chronic socioeconomic deprivation can create environments that limit an individual’s capacity for agency, for example by restricting available choices for many life decisions [57] or limiting opportunities for experiences of control [58]. The current study’s finding of an association between perceived neighborhood trust and safety and built environment conditions and cancer self-efficacy is therefore an important contribution to this relatively understudied area of research.

### Limitations

There are several limitations that must be acknowledged in the interpretation of results. First, the analysis was cross-sectional; it is therefore difficult to ascertain causality. Future research should assess neighborhood perceptions, risk perceptions and self-efficacy longitudinally but across a shorter time frame (i.e., shorter than ten-year follow-up periods) to probe causality and to assess whether associations change over time. Second, there are additional factors that affect individuals’ perceptions of both neighborhood environments and cancer risk that were not assessed in the current study. For example, individuals who tend to be pessimistic are more likely to have negative perceptions about their neighborhoods as well as their health [28]. Additionally, other individual-level factors such as emotional responses (e.g., cancer worry, anxiety, fear of positive screening findings), racial and ethnic background, acculturation, having a cancer-related symptom (e.g., a benign breast symptom), general cancer beliefs (causes, information overload), general health literacy, cancer information seeking, risk behaviors and self-reported health [13,59] have also been documented in the literature, and may be worth examination in future studies along with context. It should be noted that the associations observed in this study controlled for establishment of quality ties to others using the positive relations scale, which is a factor that may affect perceptions of both neighborhood environment and cancer risk or self-efficacy.

The present study was not a comprehensive assessment of the complex interplay of neighborhood features that affect cancer-related behaviors. In future studies examining neighborhood influences on cancer-related beliefs, attention could be given to expanding this work into other neighborhood and individual factors—for example, the interplay between neighborhood access to health-promoting resources and individual finances as related to cancer-related knowledge, behaviors and outcomes [60]. Further, although race and ethnicity and residential tenure did not moderate associations examined in the present analysis, it will be valuable for research to continue considering theory-informed moderators of associations between neighborhood-psychosocial associations. Doing so can advance thinking about how to best conceptualize how, where and for whom different neighborhood variables and individual beliefs and behaviors related to health are related to each other. There may also be neighborhood-level inequalities to consider that can impact risk, other community-level variables such as health care, quality food and physical activity resource access, employment rates and community policies could be important to examine.

Third, nuances in the way individuals perceive their cancer risk or the quality of their neighborhood may not have been captured with these brief measures. Of note, contextual factors in the current study had small unique contributions to perceived cancer risk and prevention efficacy, similar to that of other multilevel studies [61]. Investigating other factors of neighborhood quality is of importance for future research and may explain further variance in perceived cancer risk and prevention efficacy. However, it is important to note that even if environmental factors account for a small contribution to the variance explained in health-related outcomes, their effects on health at the population level may be significant and consistent over time [61]. Additionally, our stepwise approach may have underestimated variance, since sociodemographic variables were entered first and shared variance was attributed to these variables [61].

Lastly, this study used subjective measures of context and did not include objective measures. However, while examining neighborhood conditions with objective measures is of importance to capture neighborhood health effects, these are restricted by area-level boundaries and may not accurately capture community members’ definitions of “neighborhood”, exposure and experiences [30]. Weden et al. (2008) assert that subjective neighborhood measures are more proximal determinants of health [30]. Despite multiple limitations, this study had several strengths worth noting, including the use of a relatively large national sample, as well as the examination of multilevel factors. Additionally, to our knowledge this is the first study to examine perceived neighborhood conditions in relation to cancer risk perception and cancer prevention self-efficacy using a national sample.

## 5. Conclusions

Neighborhoods can influence general health outcomes through material deprivation, psychosocial mechanisms, health behaviors and access to resources. While there is conceptual acknowledgment of the importance of neighborhoods for cancer, more work can be done to examine how neighborhood context can be associated with intrapersonal factors such as perceived cancer risk [5]. Incorporating social and built environment factors into research on cancer etiology and outcomes can generate insights into disease processes, identify vulnerable populations and generate results with translational impact of relevance for interventionists and policy makers [5]. Future efforts in this area should focus on longitudinal studies to establish causal relationships and capture the effects of changes in neighborhood features, look at additional measures of environment, examine the impact of delineating geographic boundaries in different ways, assess the influence of additional individual-level factors (such as optimism) and integrate objective self-reported data regarding both the social and built environment.

## Figures and Tables

**Table 1:** Descriptive statistics for sample demographic characteristics and outcome (*N* = 2450).

	Total
Demographic Characteristics	% (*n*) or M (SD)
Age	62.14 (10.75)
Female	56.49 (1384)
**Education**	
High school degree or less	29.35 (719)
Some college	31.31 (767)
College degree	20.82 (510)
Graduate degree	18.53 (454)
**Household income**	
<$60,000	43.22 (1059)
$60,000 to $99,999	20.94 (513)
100,000+	30.57 (749)
Missing	5.27 (129)
**Married**	62.61 (1534)
**Race and ethnicity**	
Non-Hispanic White	77.76 (1905)
Non-Hispanic Black	12.69 (311)
Other	4.37 (107)
Missing	5.18 (127)
Family history of cancer	40.90 (1002)
**Residential tenure**	
<6 years	22.33 (547)
6 to 14 years	25.67 (629)
>15 years	52.00 (1274)
Reported positive relations	16.74 (3.78)
Perceived cancer prevention efficacy	5.71 (1.32)
Perceived cancer risk	2.87 (1.32)

**Notes.** Race and ethnicity combines low-frequency responses including Hispanic/Latino, Asian, Native American or Alaska Native, Native Hawaiian or Pacific Islander, and other. Family history of cancer reports on family history in immediate family only, including biological parents, brothers, sisters, or children.

**Table 2:** Standardized beta weights and 95% CI for regression analyses of perceived neighborhood conditions predicting perceived cancer risk (*N* = 2450).

	Model 1			Model 2		
	Unstandardized Estimate (SE)	95% CI	Standardized Estimate	Unstandardized Estimate (SE)	95% CI	Standardized Estimate
***Block 1***						
**Age**	−0.02 *** (0.00)	−0.03, −0.02	−0.18 ***	−0.02 *** (0.00)	−0.02, −0.01	−0.16 ***
**Female**	0.17 ** (0.05)	0.07, 0.28	0.07 **	0.18 *** (0.05)	0.07, 0.29	0.07 ***
**Education**						
≤ High school degree	__	__	__	__	__	__
Some college	−0.05 (0.07)	−0.18, 0.08	−0.02	−0.02 (0.07)	−0.15, 0.11	−0.01
College degree	−0.15 (0.08)	−0.30, −0.004	−0.05	−0.11 (0.08)	−0.26, 0.04	−0.03
Graduate degree	−0.23 ** (0.08)	−0.39, −0.07	0.07 **	−0.18 * (0.08)	−0.34, −0.02	−0.05 *
**Race and ethnicity**						
Non-Hispanic White	__	__	__	__	__	__
Non-Hispanic Black	0.15 (0.08)	−0.01, 0.31	0.04	0.09 (0.08)	−0.08, 0.25	0.02
Other	0.03 (0.13)	−0.21, 0.28	0.00	0.01 (0.13)	−0.24, 0.26	0.00
Missing	−0.03 (0.12)	−0.26, 0.20	−0.01	−0.05 (0.12)	−0.27, 0.18	−0.01
**Positive relations**	−0.02 * (0.01)	−0.03, −0.003	−0.05 *	−0.01 (0.01)	−0.02, 0.01	−0.02
**Family history of cancer**	−0.66 *** (0.05)	−0.77, −0.56	−0.25 ***	−0.66 *** (0.05)	−0.76, −0.56	−0.25 ***
**Household income**						
<$60,000	__	__	__	__	__	__
$60,000 to $99,999	−0.01 (0.07)	−0.15, 0.14	0.00	0.03 (0.07)	−0.11, 0.17	0.01
$100,000+	−0.06 (0.07)	−0.20, 0.08	−0.02	−0.02 (0.07)	−0.16, 0.12	−0.01
Missing	0.06 (0.12)	−0.17, 0.29	0.01	0.09 (0.12)	−0.15, 0.32	0.01
**Married (other, ref)**	−0.02 (0.06)	−0.14, 0.10	−0.01	0.01 (0.06)	−0.11, 0.13	0.00
**Residential tenure**						
<6 years				__	__	__
6 to 14 years				−0.18 * (0.07)	−0.33, −0.03	−0.06 *
>15 years				−0.18 ** (0.07)	−0.31, −0.04	−0.07 **
***Block 2***						
**Trust and safety**						
Tertile 1 (low)				__	__	__
Tertile 2				−0.06 (0.06)	−0.18, 0.07	−0.02
Tertile 3 (high)				−0.06 (0.08)	−0.21, 0.09	−0.02
**Social integration**						
Tertile 1 (low)				__	__	__
Tertile 2				−0.07 (0.06)	−0.20, 0.05	−0.03
Tertile 3 (high)				−0.16 * (0.07)	−0.30, −0.02	−0.05 *
**Built conditions**						
Tertile 1 (worse)				__	__	__
Tertile 2				−0.01 (0.08)	−0.17, 0.15	−0.00
Tertile 3 (better)				−0.16 * (0.06)	−0.28, −0.03	−0.06 *
**Intercept**	4.80 *** (0.20)			4.75 (0.21)	4.34, 5.15	__
**Effect modifier**						
Trust and safety X residential tenure						*p* = 0.509
Built conditions X residential tenure						*p* = 0.480
Social integration X residential tenure						*p* = 0.476
Trust and safety X race and ethnicity						*p =* 0.509
Built conditions X race and ethnicity						*p =* 0.166
Social integration X race and ethnicity						*p =* 0.408

**Notes.** 95% confidence intervals in brackets. Adjusted *R*^2^ = 9.7% for Block 1, Δ Adjusted *R*^2^ change = 0.7% for Block 2. Models 2 included perceived neighborhood exposure variables in the same model. Effect modifiers were tested in separate fully adjusted models. Boldface indicates statistical significance * *p* < 0.05, ** *p* < 0.01, *** *p* < 0.001.

**Table 3:** Standardized beta weights and 95% CI for regression analyses of perceived neighborhood conditions predicting perceived cancer prevention efficacy (*N* = 2450).

	Model 1			Model 2		
	Unstandardized Estimate (SE)	95% CI	Standardized Estimate	Unstandardized Estimate (SE)	95% CI	Standardized Estimate
***Block 1***						
**Age**	−0.01 ** (0.00)	−0.03, −0.02	−0.07 **	−0.01 ***(0.00)	−0.02, −0.01	−0.09 ***
**Female**	0.14 * (0.06)	0.07, 0.28	0.05 *	0.14 * (0.06)	0.03, 0.25	0.05 *
**Education**						
≤ High school degree	__	__	__	__	__	__
Some college	0.03 (0.07)	−0.18, 0.08	0.01	−0.00 (0.07)	−0.14, 0.13	−0.0007
College degree	0.14 (0.08)	−0.30, −0.00	0.04	0.09 (0.08)	−0.07, 0.24	0.03
Graduate degree	0.33 *** (0.08)	−0.39, −0.07	0.10 ***	0.26 ** (0.08)	0.09, 0.42	0.08 **
**Race and ethnicity**						
Non-Hispanic White	__	__	__	__	__	__
Non-Hispanic Black	0.09 (0.09)	−0.01, 0.31	0.02	0.15 (0.09)	−0.02, 0.32	0.04
Other	−0.15 (0.13)	−0.21, 0.28	−0.02	−0.12 (0.13)	−0.38, 0.13	−0.02
Missing	0.18 (0.12)	−0.26, 0.20	0.03	0.21 (0.12)	−0.02, 0.44	0.04
**Positive relations**	0.02 ** (0.01)	−0.03, −0.00	0.07 **	0.01 (0.01)	−0.01, 0.02	0.02
**Family history of cancer**	−0.05 (0.05)	−0.76, −0.56	−0.02	−0.05 (0.05)	−0.16, 0.05	−0.02
**Household income**						
<$60,000	__	__	__	__	__	__
$60,000 to $99,999	0.09 (0.07)	−0.15, 0.14	0.03	0.05 (0.07)	−0.09, 0.20	0.02
$100,000+	0.05 (0.07)	−0.20, 0.08	0.02	−0.01 (0.07)	−0.15, 0.14	−0.00
Missing	0.27 * (0.12)	−0.17, 0.29	0.05 *	0.23 (0.12)	−0.01, 0.47	0.04
**Married (other, ref)**		−0.14, 0.10	−0.03	−0.11 (0.06)	−0.23, 0.01	−0.04
***Block 2***						
**Residential tenure**						
<6 years				__	__	__
6 to 14 years				0.03 (0.08)	−0.12, 0.18	0.01
>15 years				0.02 (0.07)	−0.11, 0.16	0.01
**Trust and safety**						
Tertile 1 (low)				__	__	__
Tertile 2				0.10 (0.07)	−0.03, 0.23	0.04
Tertile 3 (high)				0.22 ** (0.08)	0.06, 0.38	0.07 **
**Social integration**						
Tertile 1 (low)				__	__	__
Tertile 2				0.20 ** (0.06)	0.07, 0.32	0.07 **
Tertile 3 (high)				0.28 *** (0.07)	0.14, 0.43	0.10 ***
**Built conditions**						
Tertile 1 (worse)				__	__	__
Tertile 2				−0.11 (0.08)	−0.28, 0.05	−0.03
Tertile 3 (better)				0.14 * (0.07)	0.01, 0.27	0.05 *
**Intercept**	5.66 *** (0.21)			5.87 *** (0.21)	5.45, 6.29	__
**Effect modifier**						
Trust and safety X residential tenure					*p* = 0.396	
Built conditions X residential tenure					*p* = 0.714	
Social integration X residential tenure					*p* = 0.746	
Trust and safety X race and ethnicity					*p =* 0.944	
Built conditions X race and ethnicity					*p =* 0.944	
Social integration X race and ethnicity					*p =* 0.059	

**Notes.** 95% confidence intervals in brackets. Adjusted R2 = 2.01% for Block 1, Δ Adjusted R2 change = 1.8% for Block 2. Model 2 included perceived neighborhood exposure variables in the same model. Effect modifiers were tested in separate fully adjusted models. Boldface indicates statistical significance * *p* < 0.05, ** *p* < 0.01, *** *p* < 0.001.

## Data Availability

The datasets generated and/or analyzed during the current study are available in the MIDUS repository, https://www.midus.wisc.edu/data/index.php (accessed on 1 August 2022).

## Supplementary Materials

The following supporting information can be downloaded at: https://www.mdpi.com/article/10.3390/ijerph21010062/s1, Table S1: Correlations between participant characteristics and perceived cancer risk and prevention efficacy (*N* = 2450); Table S2: Standardized beta weights and 95% CI for regression analyses of perceived neighborhood conditions predicting perceived cancer risk and prevention efficacy (*N* = 2450).
