# Supplementary material for: The Association of Context with Reported Self-Efficacy for Cancer-Preventive Behaviors and Perceived Cancer Risk in U.S. Adults from the Midlife in the United States (MIDUS) Study
Source: Int J Environ Res Public Health. 2024 Jan 3;21(1):62. doi: 10.3390/ijerph21010062 (PMC10815586; doi:10.3390/ijerph21010062)
Supplement: Supplementary file 1 [file ijerph-21-00062-s001.zip › ijerph-2549150-supplementary.pdf]

Table S1. Correlations between participant characteristics and perceived cancer risk and prevention efficacy (N = 2450).

	Age	Sex	Education	Income	Marital Status	Race and ethnicity	Positive relations	Family history of cancer	Residential tenure
Self-efficacy for cancer prevention	**-0.06****	**0.06****	**0.06*****	0.03	-0.01	-0.00	**0.07*****	-0.02	-0.02
Perceived cancer risk	**-0.16*****	**0.09*****	**-0.06****	-0.02	-0.04	0.02	**-0.06*****	**-0.23*****	**-0.07*****

Notes. Pearson's correlations were conducted for continuous by continuous, point biserial correlations for binary by continuous variables, Kendall's coefficient of rank correlations for continuous by ordinal, and regression test for nominal by continuous variables. Boldface indicates statistical significance. * p < 0.05, ** p < 0.01, *** p < 0.001.

Table S2. Standardized beta weights and 95% CI for regression analyses of perceived neighborhood conditions predicting perceived cancer risk and prevention efficacy (N = 2450).

	Perceived Cancer Risk			Perceived Cancer Prevention Efficacy		
	Unstandardized Estimate (SE)	95% CI	Standardized Estimate	Unstandardized Estimate (SE)	95% CI	Standardized Estimate
**Trust and safety**						
Tertile 1 (low)	—	—	—	—	—	—
Tertile 2	**-0.12* (0.06)**	-0.24, -0.002	**-0.04***	**0.18** (0.06)**	0.06, 0.30	**0.07****
Tertile 3 (high)	**-0.17* (0.07)**	-0.31, -0.03	**-0.05***	**0.36*** (0.07)**	0.21, 0.50	**0.12*****
**Social integration**						
Tertile 1 (low)	—	—	—	—	—	—
Tertile 2	-0.09 (0.06)	-0.21, 0.03	-0.03	**0.23*** (0.06)**	0.11, 0.36	**0.08*****
Tertile 3 (high)	**-0.20** (0.07)**	-0.34, -0.06	**-0.07****	**0.36*** (0.07)**	0.21, 0.50	**0.12*****
**Built conditions**						
Tertile 1 (worse)	—	—	—	—	—	—
Tertile 2	-0.04 (0.08)	-0.20, 0.12	-0.01	-0.05 (0.08)	-0.22, 0.11	-0.01
Tertile 3 (better)	**-0.19** (0.06)**	-0.31, -0.08	**-0.07****	**0.24*** (0.06)**	0.12, 0.36	**0.09*****

Notes. 95% confidence intervals in brackets. Multivariate regression analysis adjusted for age (continuous), sex, marital status (married, other [separated, divorced, widowed, never married]), education (high school degree or less, some college, college degree, graduate degree), income (<$60,000; $60,000 to $99,999; 100,000+), race and ethnicity (non-Hispanic White, non-Hispanic Black, other), well-being (continuous), family history of cancer, and residential tenure (< 6 years, 6 to 14 years, > 15 years). Models of perceived cancer risk and prevention efficacy tested main perceived neighborhood exposure variables in separate models. Effect modifiers were tested in separate fully adjusted models. Boldface indicates statistical significance. * p < 0.05, ** p < 0.01, *** p < 0.001.
